# The Importance of Plasma Renin Concentrations in Intensive Care Patients with Circulatory Shock

**DOI:** 10.3390/jcm15093184

**Published:** 2026-04-22

**Authors:** Yasemin Bozkurt Turan, Sait Karakurt

**Affiliations:** Department of Critical Care, Marmara University Pendik Training and Research Hospital, Istanbul 34899, Türkiye

**Keywords:** renin, circulatory shock, mortality, intensive care, tissue perfusion

## Abstract

**Background:** Renin is a hypoperfusion marker and a good index of renin–angiotensin–aldosterone system (RAAS) activity. The purpose of this study was to evaluate whether the plasma renin concentration (PRC) can represent a tissue perfusion marker for predicting mortality in patients with circulatory shock in intensive care. **Methods:** This prospective study included patients aged 18 years or older who were hospitalized in the intensive care unit (ICU). A total of 69 patients were enrolled, of whom 37 had circulatory shock and were all diagnosed with septic shock according to Sepsis-3 criteria, while 32 patients did not have shock. Patient groups were compared, and survival analysis was carried out. Mortality predictions of PRC, lactate and combined tests (including PRC, mottling scores, central venous saturation of oxygen, C-reactive protein, procalcitonin, and lactate) were investigated with ROC analysis. **Results:** ICU 28-day mortality was 36.2% (*n* = 25) and was significantly higher in patients with circulatory shock than those without (CS:21, 56.8% vs. NS:4, 12.5%, respectively, *p* < 0.001). The survival was significantly higher in patients without circulatory shock than those with shock (17 vs. 16 days; *p* = 0.038). The increase in mottling score (HR: 1.64 [95%CI: 1.15–2.33]; *p* < 0.01) and PRC (HR = 1.01 [95%CI: 1.00–1.02]; *p* < 0.05) levels and the decrease in glomerular filtration rate (GFR) (HR = 0.98 [95%CI: 0.96–0.99]; *p* < 0.05) were associated with decreased survival times in the ICU patients (*p* < 0.001). Combined tests yielded better prediction of mortality than PRC level alone. **Conclusions:** PRC may reflect circulatory shock and predict survival in critically ill patients; however, larger prospective studies incorporating serial PRC measurements are needed before it can be recommended as an independent biomarker of mortality.

## 1. Introduction

Shock is a state of acute circulatory failure characterized by inadequate tissue perfusion and insufficient oxygen delivery at the cellular level. It affects approximately one-third of patients in intensive care units (ICUs) [1]. There are four types of shock, distributive, cardiogenic, hypovolemic, and obstructive [2]. Among these shock phenotypes, septic shock, which belongs to the distributive shock category, is the most common type in ICU patients and remains associated with high morbidity and mortality [2,3]. If not treated promptly, irreversible organ damage, multiple organ failure, and death occur [4]. Therefore, early identification of tissue hypoperfusion and accurate prognostic stratification remain central goals in the management of critically ill patients. Several studies have shown that lactate [5], mottling scores [6], and central venous saturation of oxygen (S_cv_O_2_) [7,8] can be used in early resuscitation in sepsis and septic shock, and that their use can predict mortality.

C-reactive protein (CRP), procalcitonin, Acute Physiology and Chronic Health Evaluation II (APACHE II), and Sequential Organ Failure Assessment (SOFA) are also widely used for prognostic assessment in septic ICU patients [9,10,11].

The renin–angiotensin–aldosterone system (RAAS) is a major neurohormonal pathway involved in the regulation of vascular tone and the maintenance of circulatory homeostasis [12]. Renin is released in response to decreased renal perfusion, sympathetic activation, and reduced effective circulating volume, all of which are prominent features of septic shock [13,14]. For this reason, plasma renin concentration (PRC) may reflect both RAAS activation and the severity of hemodynamic derangement in critically ill patients [15,16].

However, despite increasing interest in renin as a biomarker, studies directly comparing PRC levels between ICU patients with and without circulatory shock remain limited [15,17]. In addition, the prognostic value of PRC alone, as compared with its use in combination with established clinical and laboratory markers, has not been fully clarified. Therefore, the aim of this study was to evaluate the relationship between PRC and circulatory shock in ICU patients and to assess its role in predicting survival and mortality, both alone and in combination with mottling score, SOFA, APACHE II, glomerular filtration rate (GFR), lactate, central venous oxygen saturation (S_cv_O_2_), C-reactive protein (CRP), and procalcitonin.

## 2. Material and Methods

### 2.1. Patients

This prospective observational study was conducted in patient groups aged 18 years and over with and without circulatory shock who were admitted to Marmara University Hospital ICU, Türkiye, between November 2019 and November 2020. Informed written consent was received either from the patients themselves or their families. The research was conducted in compliance with the Declaration of Helsinki and Good Clinical Practice guidelines. Approval was granted by the Marmara University Medical Faculty clinical research ethical committee (no. 09.2019.883).

**Figure 1:**
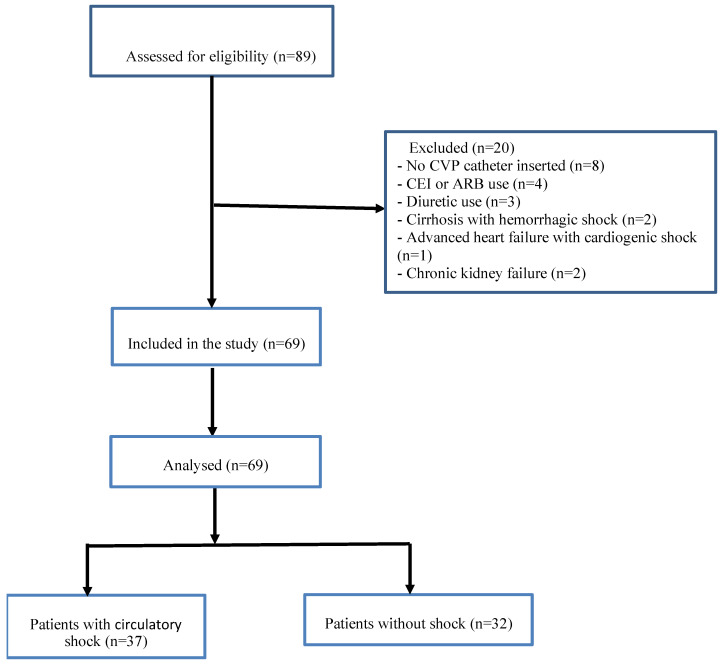
STROBE flow chart of the study participants.

Individuals with hemolytic samples, using angiotensin-converting enzyme inhibitors (ACEIs) and angiotensin receptor blockers (ARBs) in the previous 48 h, using diuretics in the previous six hours, pregnant women, lactating mothers, and patients with chronic stimulation of the mineralocorticoid axis (cirrhosis, advanced heart failure, or chronic kidney failure) were excluded from the study. Eighty-nine patients were hospitalized to intensive care during the study period, of whom eight were excluded since central venous pressure (CVP) catheters were not installed, four due to using ACEIs and ARBs in the previous 48 h and three due to using diuretics in the previous six hours were excluded. Two patients with cirrhosis with hemorrhagic shock, one with advanced heart failure with cardiogenic shock, and two with chronic kidney failure were also excluded. The study was thus performed with 69 patients, 37 in the circulatory shock group and 32 in the non-shock group (Figure 1). All patients in the circulatory shock group were diagnosed with septic shock (*n* = 37, 100%).

Strengthening the Reporting of Observational Studies in Epidemiology (STROBE) [17] instructions were applied when constituting the flow chart.

### 2.2. Specimen Collection and Measurements

Blood specimens collected for the measurement of PRC levels in patients with circulatory shock admitted to the ICU were transferred from the radial artery catheter to ethylene diamine tetra-acetic acid (EDTA) tubes. Blood specimens were also collected using the same method from the control group patients without circulatory shock.

These were centrifuged within 30 min at 2000–3000 rpm at 2–8 °C for 15 min and then stored at −80 °C for six months. Plasma renin levels were analyzed using the enzyme-linked immunosorbent assay (ELISA) method previously employed by Leśnik et al. [17]. Blood samples were obtained at a single time point in the intensive care unit within the first hour after the diagnosis of circulatory shock and before the initiation of vasoactive therapy, in order to reflect the early neurohormonal response of circulatory shock. The timing of sampling was not further standardized according to intubation status or the use of mechanical ventilation. Blood samples were not collected outside the official working hours of the laboratory. Serial measurements could not be performed due to clinical workload, logistical constraints, and the instability of critically ill patients.

Circulatory shock was defined as a systolic blood pressure below 90 mmHg, the need for inotropes or vasoactive agents to maintain systolic blood pressure at or above 90 mmHg, and clinical signs of tissue hypoperfusion (such as oliguria, mottled skin, altered mental status, cold extremities, and hyperlactatemia) [18,19]. In the non-circulatory shock group, some patients required noradrenaline for hemodynamic support despite not meeting the predefined diagnostic criteria for circulatory shock. In these patients, noradrenaline was administered for indications such as postoperative vasoplegia, sedation- or analgesia-related hypotension, or transient hemodynamic instability. However, as these patients did not fulfill the study-defined criteria for circulatory shock, they were classified in the non-circulatory shock group. Septic shock was defined according to Sepsis-3 criteria as the requirement for vasopressor therapy to maintain a mean arterial pressure ≥ 65 mmHg and a serum lactate level > 2 mmol/L despite adequate fluid resuscitation. The vasopressor treatment approach applied in the management of circulatory shock was conducted in accordance with the Surviving Sepsis Campaign 2021 Guidelines, which recommend norepinephrine as the first-line vasopressor and the addition of epinephrine in cases of inadequate hemodynamic response [20].

The patients’ demographic data; comorbidity status; vasopressor requirements; 28-day dialysis requirements; 28-day mortality rates; length of stay in the ICU; Glasgow Coma Scala (GCS); mottling score; SOFA and APACHE II scores; GFR; and lactate, S_cv_O_2_, CRP, procalcitonin, creatinine, and PRC values were recorded. GFR was calculated using the Modification of Diet in Renal Diseases Study (MDRD) formula.

### 2.3. Sample Size

The total sample size required to determine the mean difference in PRC between the circulatory shock and without shock groups was calculated as 58 (29:29), with an alpha error of 5%, power of 90%, and effect size d = 0.8, and an allocation ratio of 1:1 using G*Power version 3.1 software.

### 2.4. Statistical Analysis

Statistical analysis was conducted on IBM SPSS version 25 (IBM SPSS Statistics for Windows, Version 25.0. Armonk, NY, USA: IBM Corp.) and STATA 15 (Stata Statistical Software: Release 15. College Station, TX, USA: Stata Corp LLC.) software. Patient characteristics were compared between those with and without circulatory shock using the Independent Samples *t*-test, Mann–Whitney U test, Pearson’s Chi-Square test, Yates Continuity Correction and Fisher’s Exact test. Follow-up time was measured from the date of admission to the ICU to the date of mortality from any cause in the ICU or the date of discharge. The seven patients with survival times exceeding 40 days in the ICU were evaluated as outliers and excluded from survival analysis. Overall survival time (OST) was estimated using the Kaplan–Meier method and survival curves were compared using the Wilcoxon (Breslow) test. Parameters associated with survival were examined using the Cox Proportional Hazards (CPH) model forward stepwise method. Schoenfeld’s residuals were investigated for proportional hazards assumptions. Test results for mottling score, lactate, S_cv_O_2_, CRP, procalcitonin, and PRC were combined to improve the accuracy of 28-day mortality prediction in the ICU using Binary Logistic Regression. The area under receiver operating characteristics (ROC) curve (AUC) was used for the ICU 28-day mortality predictor. Statistical significance was set at 0.05 level.

## 3. Results

### 3.1. Patient Characteristics

The clinical characteristics and biochemical measurements of the ICU patients and comparisons between the circulatory shock and non-shock groups are shown in Table 1. The mean age of the patients was 61.5 (±16.4) years, and more than half were men (*n* = 40, 58.0%). The circulatory shock and non-shock groups were similar in terms of age, gender, Body Mass Index (BMI), and presence of comorbidity (*p* > 0.05). The use of 28-day dialysis, mechanical ventilation, terlipressin, adrenalin and noradrenalin were significantly more frequent in the circulatory shock patients (*p* < 0.01). The 28-day ICU mortality, and length of ICU stay were significantly higher in the circulatory shock patients (*p* < 0.01). The levels of SOFA, APACHE II scores, lactate, creatinine, CRP, procalcitonin, PRC, and mottling scores were significantly elevated in the circulatory shock patients than those without (*p* < 0.01). The circulatory shock patients had significantly lower three-day survival rate after discharge from the ICU, GCS, GFR, and S_cv_O_2_ levels than those without shock (*p* < 0.05) (Table 1).

### 3.2. Survival Analysis

**Figure 2:**
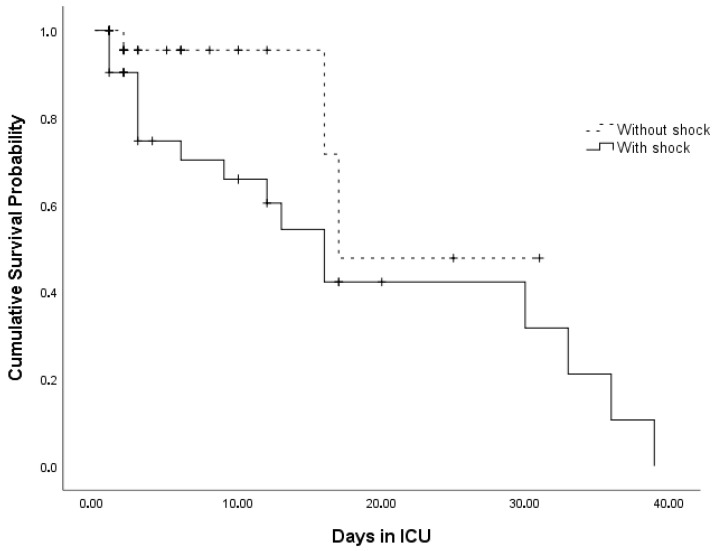
Kaplan–Meier survival curves for the circulatory shock status of patients. Cross marks indicate the time point of the event (exitus).

**Figure 3:**
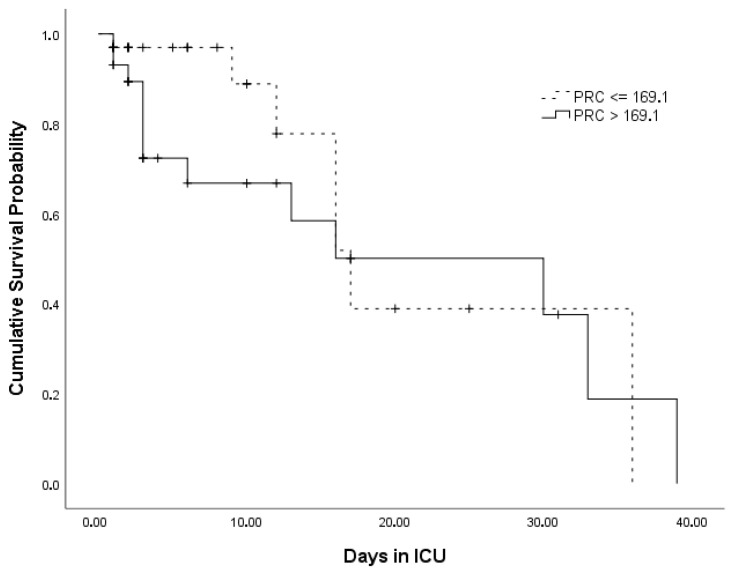
Kaplan–Meier survival curves for PRC levels. Cross marks indicate the time point of the event (exitus).

Kaplan–Meier survival curves for the circulatory shock status and PRC levels are presented in Figure 2 and Figure 3, respectively.

The median survival time was 17 [95% CI: 5.4–28.7] days, and the mortality rate among the ICU patients was 32%. The median OST was significantly higher in patients in the ICU without circulatory shock than in those with shock (Wilcoxon $\chi^{2}$ = 5.016; *p* = 0.038). The survival curves were not significantly different between the PRC levels (Wilcoxon $\chi^{2}$ = 2.774; *p* = 0.096) (Table 2).

The parameters predicting the survival of the ICU patients are shown in Table 3 (*n* = 48, number of events = 15). The increase in mottling score (HR: 1.64 [95%CI: 1.15–2.33]; *p* < 0.01) and PRC (HR = 1.01 [95%CI: 1.00–1.02]; *p* < 0.05) levels and the decrease in GFR (HR = 0.98 [95%CI: 0.96–0.99]; *p* < 0.05) were associated with decreased survival times in the ICU patients (CPH Model −2 Log Likelihood = 59,237; Chi-square = 17.105; df = 3; *p* < 0.001).

### 3.3. ROC Curve Predicting 28-Day Mortality in the ICU

#### 3.3.1. Patients with Circulatory Shock

**Figure 4:**
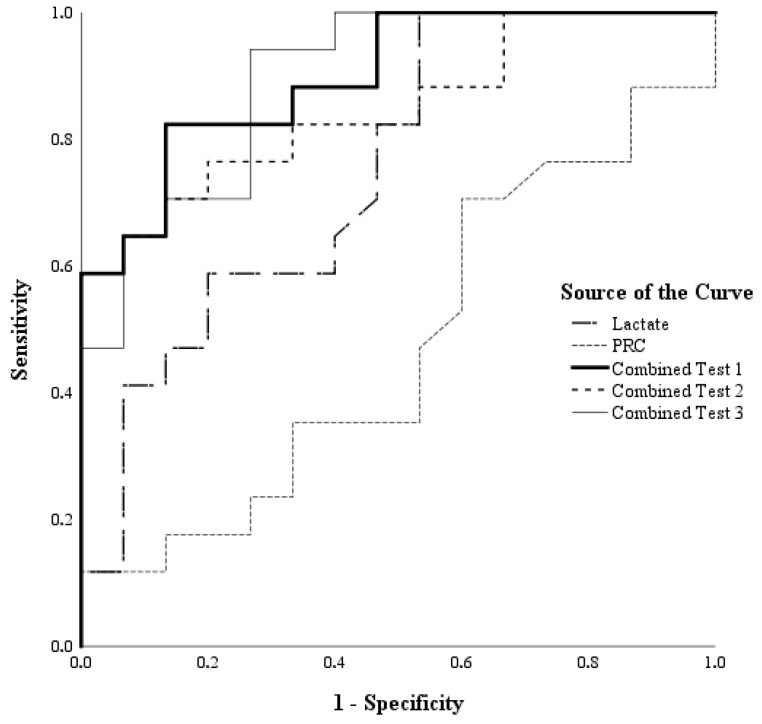
ROC curves for PRC, lactate and three combined tests predicting 28-day mortality in ICU patients with circulatory shock.

The sensitivity, specificity, and area under curves (AUC) values for PRC, lactate, and combined tests (S_cv_O_2_, CRP, procalcitonin, lactate, mottling score, and PRC) are shown in Table 4 (*n* = 32). The lactate and combined tests emerged as indicators of ICU 28-day mortality in patients with circulatory shock (*p* < 0.05), while PRC was not determined as such an indicator (*p* > 0.05). The ROC curves of the tests are presented in Figure 4.

The areas under the ROC of the five tests differed significantly (Chi-square = 21.09; df = 4; *p* < 0.001). However, there was no statistically significant difference between the AUC values for lactate and the three combined tests (Chi-square = 3.77; df = 3; *p* = 0.287). Similarly, the difference between the AUC values of the combined tests was not statistically significant (Chi-square = 1.06; df = 2; *p* = 0.589).

#### 3.3.2. Patients Without Shock

The sensitivity, specificity, and AUC values for PRC, lactate, and combined tests (S_cv_O_2_, CRP, procalcitonin, lactate, and PRC) are shown in Table 4 (*n* = 32). Combined tests 1 and 3 emerged as indicators of ICU 28-day mortality in patients without shock (*p* < 0.05), but not PRC, lactate, or Combined test 2 (*p* > 0.05). The tests’ ROC curves are shown in Figure S1. Significant differences were observed between the AUC values for PRC, lactate, and combined tests (Chi-square = 122.63; df = 4; *p* < 0.001). AUC was significantly higher for lactate than for PRC (Chi-square = 4.44; df = 1; *p* = 0.035), while no difference was determined in the AUC values for the bivariate comparisons of the other tests (*p* > 0.05).

### 3.4. Association Between PRC and Selected Clinical and Laboratory Parameters

The correlations between PRC and adrenaline, noradrenaline, terlipressin, vasopressors (adrenaline, noradrenaline and terlipressin), lactate, CRP, procalcitonin, and S_cv_O_2_ are presented in Table 5. PRC levels were not significantly associated with any of the evaluated parameters (all *p* > 0.05).

## 4. Discussion

Early identification of circulatory shock and accurate prognostic stratification remain major challenges in intensive care medicine. In the present study, PRC levels were significantly higher in patients with circulatory shock, supporting the concept that renin reflects hemodynamic stress and neurohormonal activation in critical illness. Although PRC was associated with survival in the overall cohort, it did not independently predict mortality in subgroup ROC analyses.

Univariate analysis revealed significant elevation in APACHE II scores, SOFA scores, duration of stay in the ICU, vasopressor use rates, 28-day dialysis use rates, 28-day mechanical ventilation use rates, procalcitonin levels and CRP in the patient group with circulatory shock (*p* < 0.005). These results were consistent with those of previous studies [10,11,21,22,23,24,25,26]. Similarly to Boulain et al.’s study [27], S_cv_O_2_, was significantly low in the patients with circulatory shock at univariate analysis (*p* = 0.030). However, none of these parameters were capable of predicting mortality at multivariate analysis.

In this study, the median PRC value in patients who developed circulatory shock was found to be 175.4 ng/L, and higher PRC levels were shown to be significantly associated with the development of circulatory shock (*p* = 0.001). In addition, Cox analysis performed in the overall patient cohort demonstrated that mottling score (*p* = 0.006), GFR (*p* = 0.025), and PRC (*p* = 0.038) predicted survival, and these findings were consistent with previous studies [4,15,17,27,28,29,30]. In the literature, a PRA threshold of ≥2.3 ng/mL/h has been reported to predict cardiac mortality in patients with heart failure [28], PRA ≥ 3.5 ng/mL/h to predict 28-day mortality in patients with septic shock [11], and a renin concentration cut-off value of 87 pg/mL to be associated with mortality in patients with sepsis and septic shock [17]. However, direct statistical comparison of these threshold values is not possible because these studies were conducted in different patient populations using different measurement methods. Furthermore, while PRA or renin concentration was evaluated in those studies, only PRC levels were analyzed in the present study. Direct renin concentration reflects the amount of renin present in plasma, whereas PRA reflects the enzymatic activity responsible for cleaving angiotensinogen to generate angiotensin I [31]. Indeed, elevated PRA levels have been shown to serve as a potential biomarker for predicting infection prognosis, monitoring treatment response, and following patients with septic shock [11,32,33].

Our findings support that PRC is a marker reflecting circulatory shock and survival; however, it appears to be limited in predicting mortality when used alone. It is important to interpret these results in light of the current literature. In sepsis, activation of RAAS is a complex response that plays a critical role in maintaining hemodynamic stability, while also being associated with microcirculatory dysfunction and organ injury. Indeed, in their study, Legrand et al. demonstrated that patients with elevated renin levels had a worse overall probability of survival [34]. Recent studies have provided stronger evidence supporting the role of renin in predicting mortality. In a post hoc analysis of the VICTAS Trial, higher levels of active renin were shown to be independently associated with increased mortality in critically ill patients [35]. Similarly, in a multicenter prospective study conducted by Lee et al., both plasma renin concentration and renin activity were reported to demonstrate high accuracy in predicting mortality and kidney injury in patients with septic shock [36]. Additionally, in a cohort of patients with sepsis-associated acute respiratory distress syndrome, Chakradhar et al. demonstrated that renin may serve as a novel biomarker with potential to predict in-hospital mortality [37]. In contrast, the finding that PRC did not demonstrate sufficient performance in predicting mortality when used alone in the present study shows partial discrepancy with the existing literature, and this may be explained by several important methodological and clinical factors. First, the inclusion of only patients with septic shock may have limited biological variability compared to studies involving more heterogeneous ICU populations, thereby reducing the discriminative power of the prognostic signal. Second, while previous studies have evaluated serial measurements and renin kinetics, the use of a single time-point measurement in this study may have limited the ability to capture dynamic changes and temporal physiological responses. In addition, whereas some studies have assessed both PRA and PRC, the exclusive measurement of PRC in this study may have limited the comprehensive assessment of the functional activity of the renin system.

In this study, the association between PRC and selected parameters was absent in the circulatory shock group; this situation could be due to inadequate size of sample size for correlation analysis.

Although several parameters predicting mortality have been described, since their predictive power was limited when evaluated individually, combination tests were performed based on the idea that combinations of different parameters might be more powerfully predictive.

In this study, ROC analysis performed in the patient group with circulatory shock demonstrated that multiparametric biomarker combinations (Combined Test 1, Combined Test 2, and Combined Test 3) provided higher predictive accuracy for mortality compared to single parameters such as PRC and lactate, and that this difference was statistically significant (*p* < 0.05). In the non-shock patient group, ROC analysis demonstrated that Combined Test 1 (*p* = 0.018) and Combined Test 3 (*p* = 0.042) showed significant predictive performance for mortality. These findings support a multimarker framework, in which PRC should be interpreted together with markers of microcirculation, inflammation, and organ dysfunction rather than as a standalone prognostic tool. In both shock and non-shock subgroups, combined models outperformed PRC alone, indicating that prognostic discrimination improved when renin was interpreted together with complementary markers such as lactate, CRP, procalcitonin, S_cv_O_2_, and mottling score. This observation is biologically plausible, since mortality in critical illness rarely arises from a single pathophysiological axis; rather, it reflects the interaction of hypoperfusion, inflammation, microcirculatory dysfunction, and organ failure. Consistent with the previous literature, lactate retained prognostic value in patients with circulatory shock, further supporting the benefit of combining traditional perfusion markers with PRC [5,38].

Although PRC predicted survival in the entire patient cohort in Cox analysis, it failed to predict mortality in ROC analyses of the shock and non-shock subgroups; this may have been due to the small sample size. The number of patients may have been insufficient from that perspective since the sample size was calculated using G power without mortality being predicted. The sample size was calculated for the difference in PRC levels between the groups with and without circulatory shock.

Several limitations should be acknowledged. First, PRC was measured at a single time point, which limits the ability to evaluate renin kinetics over time. Second, the limited sample size may have compromised statistical power. Third, all patients in the circulatory shock group had septic shock; therefore, the findings may not be generalizable to other shock phenotypes. Finally, because of the limited sample size, the effects of potential confounders could not be explored in greater depth.

Particular strengths of this study include its prospective design and the inclusion of a non-shock control group.

Our findings suggest that PRC is a marker reflecting circulatory shock and survival, and that it may be useful as part of a broader prognostic framework in critically ill patients. However, larger prospective studies incorporating serial PRC measurements are needed before PRC can be recommended as an independent biomarker of mortality.

## 5. Conclusions

PRC may reflect circulatory shock and predict survival in critically ill patients; however, larger prospective studies incorporating serial PRC measurements are needed before it can be recommended as an independent biomarker of mortality.

## Figures and Tables

**Table 1:** Clinical characteristics and biochemical measurements of ICU patients.

Parameter	All Patients	Patients with Circulatory Shock	Patients Without Shock	*p*-Value
Age, years, mean ± sd	61.5 ± 16.4	64.1 ± 13.8	58.5 ± 18.8	*p* = 0.162 ^a^
Male, *n* (%)	40 (58.0%)	24 (64.9%)	16 (50.0%)	*p* = 0.212 ^b^
BMI, median (IQR) kg/m^2^	27.4 (23.5–31.2)	27.2 (23.4–30.8)	27.7 (24.0–31.3)	*p* = 0.521 ^c^
28-day dialysis use, *n* (%)	14 (20.3%)	13 (92.9%)	1 (7.1%)	*p* = 0.002 ^b^*
28-day mechanical ventilation use, *n* (%)	32 (46.4%)	28 (87.5%)	4 (12.5%)	*p* < 0.001 ^b^*
Diagnosis, *n* (%)				
Surgical, *n* (%),	16 (23.2%)	3 (18.8%)	13 (81.2%)	*p* = 0.001 ^b^*
Malignancy, *n* (%),	25 (36.2%)	12 (48.0%)	13 (52.0%)	
Respiratory distress, *n* (%)	14 (20.3%)	10 (71.4%)	4 (28.6%)	
Other diseases, *n* (%)	14 (20.3%)	12 (85.7%)	2 (14.3%)	
Comorbidity, *n* (%),	52 (75.4%)	29 (55.8%)	23 (44.2%)	*p* = 0.584 ^b^
Hypertension, *n* (%),	33 (47.8%)	18 (54.5%)	15 (45.5%)	*p* = 0.999 ^b^
Diabetes mellitus, *n* (%)	17 (24.6%)	10 (58.8%)	7 (41.2%)	*p* = 0.781 ^b^
Cardiovascular diseases, *n* (%),	21 (30.4%)	11 (52.4%)	10 (47.6%)	*p* = 0.999 ^b^
Respiratory disease, *n* (%)	2 (2.9%)	1 (50.0%)	1 (50.0%)	*p* = 1.000 ^d^
Malignancy, *n* (%)	6 (8.7%)	3 (50.0%)	3 (50.0%)	*p* = 1.000 ^d^
Other diseases, *n* (%)	6 (8.7%)	4 (66.7%)	2 (33.3%)	*p* = 0.809 ^e^
Terlipressin use, *n* (%)	7 (10.1%)	7 (100.0%)	0 (0.0%)	*p* = 0.028 ^e^*
Adrenaline use, *n* (%)	12 (17.4%)	12 (100.0%)	0 (0.0%)	*p* < 0.001 ^e^*
Noradrenaline use, *n* (%)	38 (55.1%)	37 (97.4%)	1 (2.6%)	*p* < 0.001 ^d^*
28-day mortality, *n* (%)	25 (36.2%)	21 (56.8%)	4 (12.5%)	*p* < 0.001 ^b^*
Three-day survival after discharge from the ICU, *n* (%)	46 (66.7%)	17 (45.9%)	29 (90.6%)	*p* < 0.001 ^b^*
Length of ICU stay in days, median (IQR)	6.0 (2.0–16.5)	10.0 (3.0–31.5)	3.0 (1.0–9.5)	*p* = 0.006 ^c^*
GCS, median (IQR)	13.0 (3.5–15.0)	6.0 (3.0–14.5)	15.0 (13.3–15.0)	*p* < 0.001 ^c^*
SOFA, median (IQR)	5.0 (2.0–11.0)	11.0 (6.5–13.0)	1.5 (0.0–4.0)	*p* < 0.001 ^c^*
APACHE II, mean ± sd	16.1 ± 7.2	18.5 ± 7.1	13.4 ± 6.5	*p* = 0.003 ^a^*
GFR, median (IQR) mL/min/1.73 m^2^	79.7 (36.2–119.9)	42.6 (30.9–103.3)	92.7 (68.5–131.4)	*p* = 0.006 ^c^*
Lactate, median (IQR) mmol/L	1.9 (1.3–4.7)	3.0 (1.5–5.8)	1.5 (1.0–2.6)	*p* < 0.001 ^c^*
S_cv_O_2_, mean ± sd %	70.9 ± 11.7	68.1 ± 12.7	74.2 ± 9.7	*p* = 0.030 ^a^*
Creatinine, median (IQR) mg/dL	0.9 (0.7–1.8)	1.6 (0.7–2.1)	0.8 (0.6–1.0)	*p* = 0.003 ^c^*
CRP, median (IQR) mg/L	99.6 (35.4–198.0)	153.0 (73.5–259.0)	49.8 (13.0–106.3)	*p* < 0.001 ^c^*
Procalcitonin, median (IQR) µg/L	1.6 (0.3–9.3)	5.3 (1.8–22.9)	0.3 (0.1–0.9)	*p* < 0.001 ^c^*
PRC, median (IQR) ng/L	169.1 (151.5–196.4)	175.4 (162.6–234.8)	155.4 (147.8–175.6)	*p* = 0.001 ^c^*
Mottling score, median (IQR)	0.0 (0.0–1.0)	0.5 (0.0–2.0)	0.0 (0.0–0.0)	*p* < 0.001 ^c^*

Patients with and without shock were compared between *n* = 37 versus *n* = 32, respectively (excluding for BMI *n* = 32 versus *n* = 26 and Mottling score *n* = 32 versus *n* = 29). ^a^ Independent Samples *t*-test reported with mean ± SD; ^b^ Pearson Chi-Square test reported with frequency (%); ^c^ Mann–Whitney U test reported with median (interquartile range); ^d^ Fisher’s Exact and ^e^ Yates continuity correction tests reported with frequency (%). * Statistically significant at 0.05 level.

**Table 2:** Survival and mortality descriptives of ICU patients.

	Number of Events /Total Group	Median OST [95% CI]	Mortality Rate [95% CI]	*p*-Value
All patients	20/62	17.0 [5.4–28.7]	0.32 [0.24–0.40]	
Circulatory shock	17/31	16.0 [11.1–20.9]	0.55 [0.36–0.74]	0.038 ^a^*
No shock	3/31	17.0 [NC]	0.10 [0.06–0.13]	
PRC ≤ 169.1	7/33	17.0 [12.7–21.3]	0.21 [0.14–0.28]	0.096 ^a^
PRC > 169.1	13/29	30.0 [7.0–53.0]	0.45 [0.29–0.61]	

NC: Standard error and 95% CI are not computed. ^a^ Wilcoxon (Breslow) test *p*-value comparing survival curves. * Statistically significant at 0.05 level.

**Table 3:** Cox proportional hazard model predicting survival in ICU patients.

Parameter ^a^	Hazard Ratio [95% CI]	*p*	Test of Proportional Hazards Assumption		
			Chi-Square	df	*p* ^b^
Mottling score	1.64 [1.15–2.33]	0.006	0.48	1	0.487
GFR	0.98 [0.96–0.99]	0.025	0.82	1	0.365
PRC	1.01 [1.00–1.02]	0.038	0.05	1	0.817

^a^ Variable selection with the forward (likelihood ratio) stepwise method (*n* = 48). The variables entered the model were age, gender, BMI, mechanical ventilation use, dialysis use, GCS, SOFA, APACHE II, mottling score, GFR, lactate, S_cv_O_2_, creatinine, CRP, procalcitonin, PRC, and presence of circulatory shock. ^b^ Schoenfeld residuals test *p*-value.

**Table 4:** Performance of the tests predicting 28-day mortality in ICU patients with and without circulatory shock.

With circulatory shock							
Test	AUC	SE	95% CI for AUC	*p* ^a^	Sensitivity	Specificity	Accuracy
PRC ^b^	0.435	0.098	0.225–0.607	0.503	70.00%	35.29%	54.05%
Lactate ^c^	0.731	0.085	0.517–0.859	0.017	95.00%	47.06%	72.97%
Combined test 1 ^d^	0.898	0.053	0.794–0.999	<0.001	82.35%	80.00%	81.25%
Combined test 2 ^e^	0.847	0.069	0.713–0.981	0.001	85.71%	70.59%	78.13%
Combined test 3 ^f^	0.894	0.055	0.786–0.999	<0.001	58.82%	93.33%	75.00%
Without shock							
Test	AUC	SE	95% CI for AUC	*p* ^a^	Sensitivity	Specificity	Accuracy
PRC ^g^	0.397	0.183	0.038–0.755	0.561	66.67%	44.83%	46.88%
Lactate ^h^	0.540	0.221	0.106–0.974	0.821	66.67%	72.41%	71.87%
Combined test 1 ^i^	0.920	0.064	0.794–0.999	0.018	33.33%	96.55%	90.63%
Combined test 2 ^j^	0.678	0.153	0.378–0.979	0.316	33.33%	100.00%	93.75%
Combined test 3 ^k^	0.862	0.119	0.628–0.999	0.042	66.67%	100.00%	96.88%

^a^ Null hypothesis: true area = 0.5. ^b^ cut-off = 169.34. ^c^ cut-off = 1.50. ^d^ PRC, Mottling score, S_cv_O_2_, CRP, and Procalcitonin. ^e^ PRC, Lactate and Mottling score. ^f^ PRC, Lactate, Mottling score, CRP, and Procalcitonin. ^g^ cut-off = 154.61. ^h^ cut-off = 2.40. ^i^ PRC, S_cv_O_2_, CRP and Procalcitonin. ^j^ PRC and Lactate. ^k^ PRC, Lactate, CRP, Procalcitonin.

**Table 5:** Correlation between PRC and selected parameters.

With Shock								
	Use of Adrenaline ^a^	Use of Noradrenaline ^a^	Use of Terlipressin ^a^	Use of Vasopressors ^a^	Lactate ^b^	S_cv_O_2_ ^b^	CRP ^b^	Procalcitonin ^b^
PRC	r = −0.18 *p* = 0.283	^1^ NC	r = −0.29 *p* = 0.077	^1^ NC	r = 0.20 *p* = 0.244	r = 0.05 *p* = 0.758	r = −0.06 *p* = 0.744	r = 0.27 *p* = 0.113
Without Shock								
	Use of Adrenaline ^a^	Use of Noradrenaline ^a^	Use of Terlipressin ^a^	Use of Vasopressors ^a^	Lactate ^b^	S_cv_O_2_ ^b^	CRP ^b^	Procalcitonin ^b^
PRC	^2^ NC	r = −0.06 *p* = 0.751	^2^ NC	r = −0.06 *p* = 0.751	r = −0.39 * p = 0.027	r = −0.20 *p* = 0.264	r = 0.59 * p < 0.001	r = 0.23 *p* = 0.212

NC: not computed. ^1^ Use of noradrenaline and vasopressors were present in all CS patients, while ^2^ use of adrenaline and terlipressin were absent in all NS patients. ^a^ Rank biserial correlation and ^b^ Spearman Rank correlation coefficients are presented. * Statistically significant at 0.05 level.

## Data Availability

The datasets used and/or analyzed during the current study are available from the corresponding author on reasonable request.

## Supplementary Materials

The following supporting information can be downloaded at: https://www.mdpi.com/article/10.3390/jcm15093184/s1, Figure S1. ROC curves of PRC, lactate and combined tests in predicting ICU 28-day mortality in patients without shock.
