# Supplementary material for: The Importance of Plasma Renin Concentrations in Intensive Care Patients with Circulatory Shock
Source: J Clin Med. 2026 Apr 22;15(9):3184. doi: 10.3390/jcm15093184 (PMC13163410; doi:10.3390/jcm15093184)
Supplement: Supplementary file 1 [file jcm-15-03184-s001.zip › jcm-4183101-supplementary.pdf]

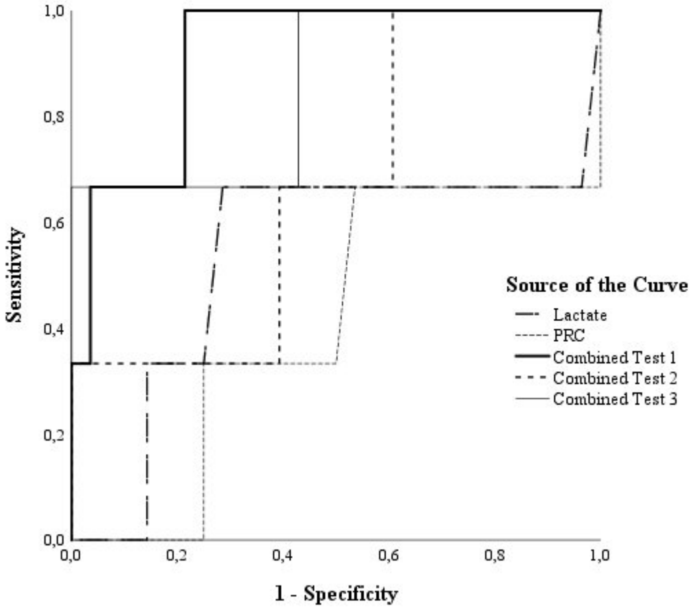

Figure S1. ROC curves of PRC, lactate and combined tests in predicting ICU 28-day mortality in patients without shock.
